# 

**Published:** 1854-02

**Authors:** 


Our Exchanges and Correspondents are particularly requested to be careful to direct to the "Iowa Medical Journal, Box 109, Keokuk, Iowa." This precaution, especially the number of the box, has become peculiarly important, as we have reason to believe that some of our valuable exchanges have unfortunately reached a destination quite different from that intended.

